# Clinicodemographic Profile and Clinical Outcome of Children Presenting to Telemedicine Center at Institute of National Importance of India: A Prospective Observational Study

**DOI:** 10.1155/2024/5341988

**Published:** 2024-01-31

**Authors:** N. Yankappa, Anil Kumar, Arun Prasad, Lokesh Tiwari, Pradeep Kumar

**Affiliations:** ^1^Department of Paediatrics, All India Institute of Medical Sciences, Patna, India; ^2^Department of Trauma Surgery & Critical Care, All India Institute of Medical Sciences, Patna, India

## Abstract

**Background:**

There is a global shortage of healthcare professionals, especially in developing countries, leading to disparities in access to healthcare, worsened by the pandemic. Telemedicine is emerging as a solution, with growing adoption worldwide due to advancements in technology and increased awareness. *Research Problem*. The establishment of telemedicine depends on resources, infrastructure, and knowledge about healthcare needs. Further studies are needed to monitor and address evolving issues in telemedicine. *The Overall Purpose of the Study*. Rural health disparities stem from multiple factors, like limited healthcare access, workforce shortages, lifestyle choices, and lower socioeconomic status, leading to higher mortality and chronic diseases. Addressing these challenges is vital for rural community well-being. Telemedicine centers present a promising solution, bridging gaps, and improving healthcare outcomes for underserved remote populations. *Methodology*. Objective: This study assessed the clinicodemographic profile and clinical outcome of children presenting to the telemedicine center at the Institute of National Importance in India. Design: Prospective observational study. Setting: A single-center tertiary care level. Participants: This study included 79 children aged up to 18 years. *Major Findings and Summary of Interpretations*. In our study, 79 children using telemedicine found a near-equal gender distribution. 8.9% needed emergency care, with common complaints being respiratory issues, fever, abdominal pain, and vomiting. After two weeks, 83.5% showed improvement, emphasizing telemedicine's effectiveness in pediatric care.

**Conclusion:**

Our study underscores telemedicine's positive impact on pediatric healthcare, emphasizing its potential to enhance access, outcomes, and cost-efficiency. Wider telemedicine adoption can reduce morbidity and mortality, support preventive care, and streamline posttreatment services, alleviating pressure on specialized facilities. While our focus was pediatrics, the telemedicine model is adaptable to various age groups and conditions, but it should be seen as a valuable supplement to, not a total substitute for, in-person healthcare visits.

## 1. Introduction

There is a global shortage of healthcare professionals, especially in developing countries [1, 2]. Rural communities experience significant health disparities, such as elevated mortality rates, decreased life expectancy, and a higher prevalence of chronic diseases. These disparities arise from a multifaceted web of factors, including limited access to healthcare services, challenges in recruiting qualified healthcare providers, lifestyle and behavioral choices, and lower socioeconomic status. During the COVID pandemic, the problem was exacerbated due to restrictions on in-person visits to clinics [3]. To uplift the health and well-being of rural areas, it is crucial to tackle these complex challenges head-on.

To address these issues, telemedicine emerged as an alternative, and its use has grown worldwide due to advancements in technology and increased awareness [4]. The establishment of telemedicine centers (TMC) emerges as a promising solution to bridge healthcare disparities in remote regions, leading to better patient outcomes and facilitating ongoing care for those with limited resources.

The first telemedicine was installed in Boston in 1967 [5]. Telemedicine, particularly through the software application which was user friendly has been effective in providing healthcare services in outreach areas [6]. The study is aimed at assessing the effectiveness of telemedicine in treating the pediatric population in such settings, and with adequate training, it can improve the understanding of patients' clinical and demographic profiles.

### 1.1. What Is Already Known on This Topic

The concept of telemedicine has been in existence for more than half a century, but it came into practice only after the advancement and widespread availability of the internet in remote areas for the last couple of years.

### 1.2. What This Study Adds

This study is the first of its kind in the state of Bihar and the eastern region of India. A national institute is connected directly to individuals of remote areas in multiple peripheral TMCs for healthcare advice, especially after the advancement in the field of information technology.

### 1.3. How This Study Might Affect Research, Practice, or Policy

Disseminating the concept of telemedicine across the country will reduce morbidity and mortality, as well as be helpful in primordial and primary prevention by telehealth education, secondary prevention by early diagnosis and treatment (Including referral advice), and tertiary prevention by helping in follow-up care. This policy will be cost-effective, time-saving, helpful in reducing the burden on tertiary centers, and easy to implement across the country. On the prototype of our study on the pediatric age group, we can replicate such a concept for geriatric, adult, obstetric, and chronic illnesses.

## 2. Methods

### 2.1. Participants

In our study, we established specific inclusion criteria, which encompassed children up to the age of 18 who were seeking care at the peripheral TMCs associated with the All India Institute of Medical Sciences (AIIMS), Patna. We excluded data about reports, investigations, or presenting complaints that could be provided by parents or caregivers in the absence of the child's physical presence.

### 2.2. Data Collection

A qualified staff member conducted a face-to-face interview with the child, involving their parents as well, to collect their primary data. Subsequently, this data was transmitted to AIIMS Patna TMC using software applications such as EVOLKO, Skype, or other internet-based mobile applications. The principal investigator made weekly visits to AIIMS Patna TMC to gather patient data shared by peripheral TMCs. During these routine visits, the data was abstracted and entered into a designated proforma as part of the process.

### 2.3. Data Variables

The study examines several independent variables, including age, sex, comorbidities, and presenting complaints. The observed outcome variables encompass symptom resolution (considered as “cured”) and symptom deterioration (termed as “not cured”). Additionally, potential confounding and interacting variables, such as disease duration, the economic status of patients, the educational background of patients and their guardians, as well as technical issues, may exert an influence on the study.

### 2.4. Study Design

**Figure 1:**
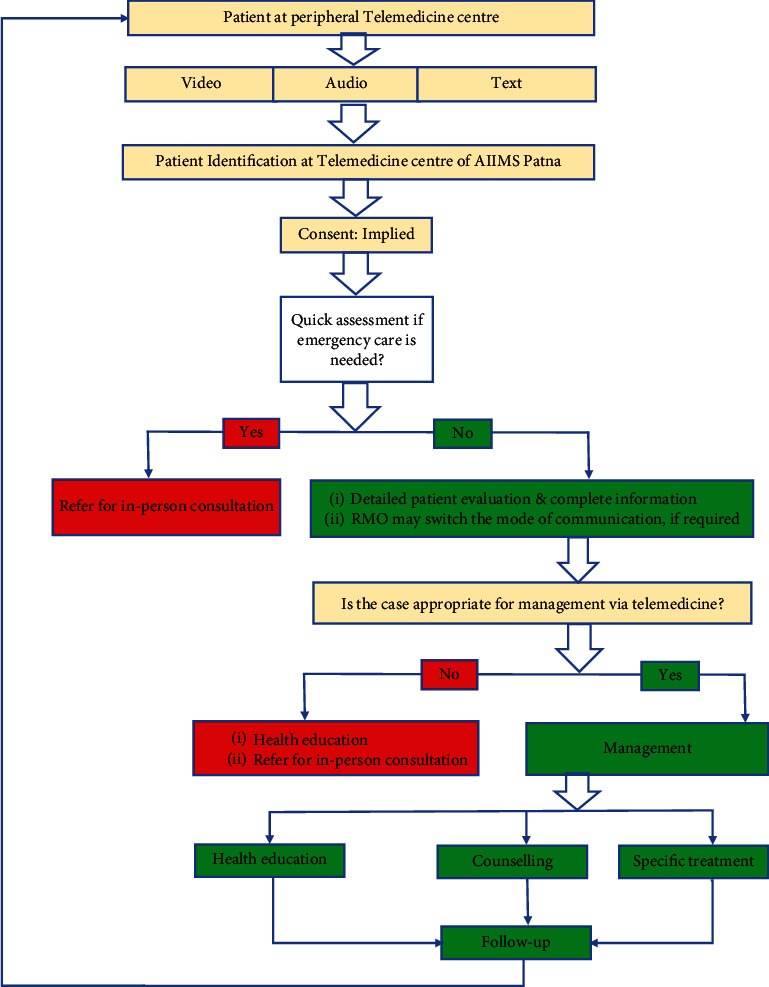
Method of teleconsultation.

The responsible staff members at the respective peripheral TMC were trained to register all the children who sought teleconsultation services. These registrations were carried out using software applications such as “EVOLKO,” Skype, or other internet-based mobile applications, and the data was promptly shared with the TMC at AIIMS, Patna, in real-time. At the TMC, a dedicated registered medical officer (RMO), consultant pediatrician, or pediatric resident conducted online consultations using convenient communication methods. Telemedicine was utilized to address the patient's needs by institutional protocols. Patients were advised to schedule follow-up sessions at the TMC based on their requirements, as illustrated in Figure 1. Additionally, the principal investigator conducted telephonic follow-ups with the patients one week and two weeks after their telemedicine consultation to evaluate the progress of their clinical condition. In our study, patients were considered cured if their symptoms were completely resolved, as reported by either the caregiver or the patient.

### 2.5. Statistical Analysis

Data analysis was conducted with IBM SPSS (Statistical Package for Social Sciences) version 22.0. Proportions were used to describe nominal data, while mean and standard deviation were employed for normally distributed discrete data. Skewed discrete data was characterized using the median and interquartile range. The statistical significance threshold was set at a *p* value of less than 0.05.

### 2.6. Ethical Approval

The research proposal was approved by the ethics committee of AIIMS, Patna, with reference number AIIMS/Pat/IEC/PGTh/Jan20/32.

## 3. Results

**Figure 2:**
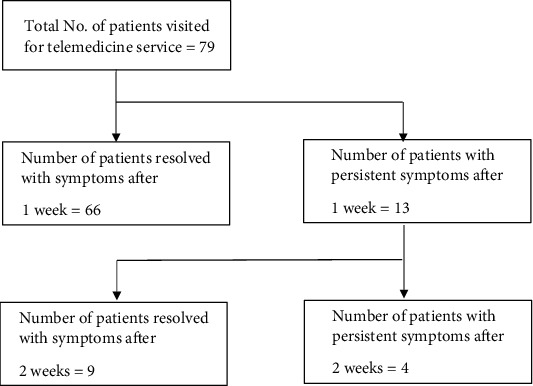
Study flowchart.

This study spanned from June 2021 to May 2022 and observed fluctuations in case numbers. The highest number of cases occurred in September 2021 (28), followed by October 2021 (19) and July 2021 (13). A total of 79 children participated in this study (Figure 2). Among them, 41 (51.9%) were female, and 38 (48.1%) were male. The average age of the children was 116.3 months, with a standard deviation (SD) of 69.9 and a range from 3 months to 18 years. Notably, 8 (10.1%) patients had a history of chronic illness, but none of them had recent hospitalization (Table 1).

During telemedicine consultations, 7 children (8.9%) needed emergency care and were sent to the nearest specialized center. Our study found that children requiring emergency care during telemedicine consultations had a significantly higher risk of their symptoms getting worse compared to those who did not need emergency care (relative risk (RR) with 95% confidence interval (CI): 9.3, 1.7-48.6) (Table 2).

**Figure 3:**
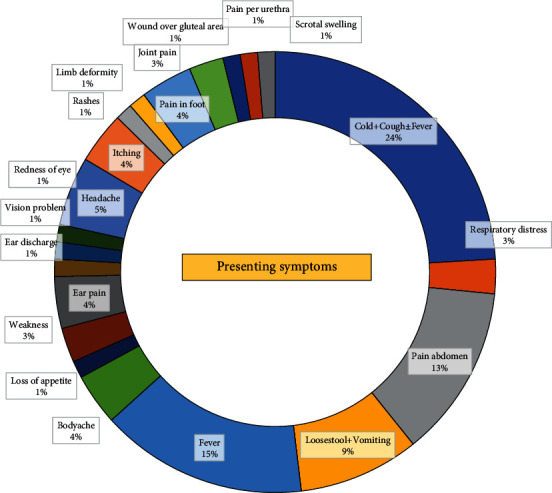
Clinical features of children presenting to TMC (telemedicine center).

The most common complaints among the children were related to respiratory symptoms (24%), followed by fever (15%), abdominal pain (13%), and vomiting with loose stools (9%) (Figure 3). All children received health education during teleconsultation. Among the 79 children, 46 were treated at the initial center and sent home, while the remaining 33 were referred to higher centers. Out of those referred, 2 did not receive treatment advice due to the seriousness of their condition, 5 received basic treatment and a referral recommendation from the doctor, and 26 could have been managed at the initial center but were taken to higher centers by their caregivers. Our study found that patients referred for specialized treatment had a significantly higher risk of their symptoms getting worse compared to those who did not need specialized treatment referral (RR with 95% CI: 6.2, 1.5-24.9).

One week after the telemedicine consultation, 66 children (83.5%) saw their symptoms resolve, which was considered a sign of recovery. The remaining 13 children still had symptoms or had symptoms that were getting worse, so they were followed up after 2 weeks. After 2 weeks, 9 more children showed improvement in their symptoms, while 4 children still had clinical symptoms.

## 4. Discussion

### 4.1. Development of Telemedicine Services

Distance poses a significant obstacle to delivering optimal healthcare services [7]. In our study, the majority of telemedicine consultations were provided to children residing in remote areas, far from urban centers. To ensure the sustainability of telehealth services, integration into routine practice is essential, enabling the provision of successful services in remote and rural areas [8].

### 4.2. Telemedicine during SARS-CoV-2 Pandemic

The COVID-19 pandemic has fully harnessed the potential of telemedicine during these unprecedented times, even though its use in the Indian healthcare system has been sporadic up to this point [9]. In our study, the majority of telemedicine services were provided during the second wave of the COVID-19 outbreak. Factors such as affordability, availability, language accommodation, and accessibility through smartphones and high-speed internet have significantly improved the acceptance of telemedicine during the pandemic [10, 11]. Moreover, with its advantages as a logical extension of healthcare's technical and technological aspects and its cost-effectiveness, telemedicine may soon become an integral part of healthcare organizations [12].

Walters et al. conducted a study during the COVID-19 pandemic to assess the feasibility of telemedicine for both acute and chronic care, with 62% of telemedicine visits addressing acute concerns and 38% for chronic conditions [13]. In our study, 71 (89.9%) patients presented with acute illnesses, while 8 (10.1%) children had known chronic illnesses during telemedicine consultations. A significant number of patients sought assistance for respiratory illnesses, likely due to increased awareness of serious viral illnesses like COVID-19.

A follow-up study by Nogueira López et al. reported that out of 72 children with suspected COVID-19 infection, 19 (26.4%) experienced clinical worsening during follow-up through teleconsultation [14]. In contrast, our study found that only 4 (5%) children remained symptomatic (unrelated to COVID-19 illness) by the end of the second week of telemedicine consultations. Agarwal et al. highlighted the importance of telemedicine in the era of the COVID-19 pandemic, concluding that while it cannot replace in-person consultations or emergency medicine, its adoption by more healthcare practitioners and patients in various forms can make a substantial contribution to managing the pandemic [15].

The successful clinical outcomes of community care under the concept of telemedicine during COVID-19 were observed in Bihar State [16]. Similarly, in our study, telemedicine services proved to be cost-effective during the pandemic, facilitating early access to healthcare services for remote populations. Therefore, telemedicine should also be considered in postpandemic healthcare models [17].

### 4.3. The Utilization of Internet-Based Mobile Applications in the Provision of Telemedicine Services

Mangwi Ayiasi et al. conducted a community intervention trial to investigate the impact of mobile phone-assisted village health team (VHT) home visits on maternal and newborn care practices. The study yielded positive results, as it facilitated timely healthcare seeking for facility-based deliveries and addressing newborn illnesses by addressing patient concerns and referrals [18]. In our research, patients received treatment recommendations based on information they shared via mobile-based applications such as text messages, WhatsApp, Skype, and EVOLKO. We also maintained telephonic communication with these patients during follow-up to assess their clinical progress. Our study revealed that mobile phones proved to be effective and user-friendly devices for both peripheral and central TMC.

### 4.4. Impact of Telemedicine Services on Clinical Outcome

Lapointe et al. conducted a systematic review to investigate the influence of telemedicine on the clinical management of trauma patients in rural areas [19]. While their study findings suggested that telemedicine may enhance the diagnosis and management of patients, its impact on mortality and complications in rural trauma cases was found to be minimal. In the medical field, researchers employ big data not only to identify disease risk factors but also to assist physicians in accurately diagnosing patients' illnesses and conditions [20]. Telecardiology, a valuable resource in resource-stretched developing countries with a majority of rural patients and urban-centered specialists, has demonstrated effective care with no significant diagnostic errors [21]. In our study, we did not utilize modern technologies like digital stethoscopes and digital otoscopes due to the self-setting of peripheral telemedicine centers by trained staff. The incorporation of these modern tools may further enhance the effective management of patients. Telemedicine has significantly improved the time to follow-up after hearing screening when used for mobile health screening and referral, as indicated in previous research [22].

Singh et al. conducted a cross-sectional study to assess the impact of telemedicine on healthcare services, evaluating patient satisfaction and identifying barriers [23]. Their findings revealed that telemedicine consultations influenced diagnosis in 39.1% of patients and aided in clinical management for 58.3%. Specialist consultations were the most common reason for teleconsultations in the public healthcare system. Barriers to teleconsultation included the lack of in-person contact and user-unfriendly software. In contrast, our study involved consultant pediatricians to manage children presented at the telemedicine center of AIIMS Patna whenever necessary. Proper triage allocation of children at the time of presentation to the telemedicine center has also demonstrated a positive impact, with sick patients being referred urgently for definitive care.

### 4.5. Challenges to Telemedicine Services

Uma Rani et al. conducted a study to assess the operational aspects of telemedicine and to analyze the best practices and challenges faced by Apollo Hospitals in providing telemedicine services [24]. The study revealed that 60% of the rural population found telemedicine beneficial, particularly in remote areas, and 70% of patients reported a reduction in the overall cost of medical care. On August 9, 2020, the Government of India launched a telemedicine service called eSanjeevani as part of the “Digital India” initiative, enabling primary healthcare centers to receive teleconsultations from medical colleges and large government hospitals in the states [25]. However, the quality of services was compromised in some states due to a shortage of available doctors [26]. Similar services were also offered in our institute, with dedicated resident medical officers (RMOs), potentially impacting the number of patients seeking teleconsultations at our peripheral telemedicine center (TMC).

A cross-sectional study conducted by Kustwar and Ray is aimed at assessing the perceptions of patients and doctors regarding telemedicine in Apollo Telehealth Services in India. The study included 122 participants, consisting of 71 patients and 51 doctors. Results indicated that 80% of patients and all doctors were satisfied with telemedicine consultations, and 90% of participants found it cost-effective. Furthermore, 61% of doctors reported an increase in the number of patients seeking teleconsultations [27]. In our study, all parents of children who underwent telemedicine consultations expressed satisfaction with the services provided. However, it is worth noting that if we have access only to an older computer or a slow internet connection, the process can become time-consuming and laborious [28].

While telehealth is expected to become more familiar and useful, people's attitudes toward telemedicine are significantly influenced by privacy concerns and outcome beliefs [29, 30, 31]. Additionally, in remote areas, accessing prescribed medication can be challenging due to its nonavailability, which was also a concern in our study due to the lack of well-established medical stores in proximity to the rural population. To this day, patients in remote areas often have to travel long distances from their homes or villages to access teleconsultation facilities [32]. There were many challenges of telemedicine services including the digital divide, privacy and security concerns, regulatory barriers, diagnostic limitations, technological infrastructure, resistance to change the people as well as health professionals, and nonavailability of prescribed medicine in the remote area. On the positive side, enabling factors such as advancements in technology, telecommunication infrastructure, remote monitoring devices, the impact of the COVID-19 pandemic, evolving regulations, and increased acceptance will contribute to the dissemination of the concept of telemedicine across the world in the coming days.

### 4.6. Follow-Up

Telemedicine is widely acknowledged worldwide for its established value in various subspecialties such as cardiology, dermatology, and neurology [33]. Utilizing online mobile applications for scheduled follow-up appointments offers an efficient means to enhance the likelihood and effectiveness of follow-up care while reducing missed appointments and optimizing patient outcomes [34, 35]. In our study, we observed that 94.9% of children exhibited clinical improvement by the end of the second week of telemedicine consultations. Consequently, we can deduce that patient follow-up care represents a crucial component in enhancing the effectiveness of telemedicine services.

### 4.7. Prospects for the Future of Telemedicine Services

The popularity of telemedicine services has been on the rise, especially in the postpandemic era. This surge in popularity can be attributed to its easy accessibility, affordability, and growing awareness among the general population. In our modern world, with the continuous advancement of information technology and its application in the field of telemedicine, online consultations have seen a rapid increase [36, 37]. With advanced technologies in this digitized age, telemedicine is expanding its reach across various healthcare sectors, including rehabilitation and the ongoing care of chronic illnesses among individuals of all age groups [38, 39, 40].

Furthermore, telemedicine services are not limited to rural and remote areas; they are also gaining traction among urban populations due to increased awareness and educational attainment. In their busy daily lives, people are increasingly drawn to time-saving methods like teleconsultations over in-person appointments, as they can receive medical care at their convenience. The real-time interaction, meticulous documentation of details, and easy reproducibility of health-related data make telemedicine even more convenient in today's circumstances.

A meta-analysis conducted by Kraef et al. revealed moderate evidence of improved disease control with digital telemedicine [41]. Data-driven healthcare has the potential to transform healthcare delivery and enhance patient outcomes. Nevertheless, it is essential to comprehensively evaluate and address the inherent challenges and risks associated with this approach [42].

Notably, healthcare professionals have had ethical concerns, including instances of blackmail, defamation, hate speech, false accusations in court, and privacy violations. A significant percentage of Indian physicians (39.2%) and Egyptian physicians (24%) believe that the penalties for such misconduct should be less severe in the context of telemedicine compared to traditional practice [43].

With further development of robust privacy policies, medicolegal guidelines, and the utilization of advanced technologies, along with proper training for providing care via telemedical platforms, it is unsurprising that telemedicine services may become one of the standard approaches to healthcare issues, alongside traditional in-person consultations [44, 45]. The concept of telemedicine continues to evolve in the present era, and in the future, it will be essential to incorporate the concept of medicolegal implications to ensure the resilience and effectiveness of telemedicine services [46, 47].

### 4.8. Limitations

Despite including all the children presented to the TMC of AIIMS Patna through its peripheral TMC, the required sample size was small. This may be attributed to the lower prevalence of pediatric patient visits to the TMC and the absence of emergency services in our peripheral TMC. Since the peripheral TMC serves as a mediator between the patient and the central TMC of AIIMS Patna, there are no facilities available for investigations, medications, or any other health services for the patients.

## 5. Conclusion

In conclusion, our study highlights telemedicine's effectiveness in improving pediatric healthcare, with potential benefits for accessibility and outcomes. Widespread telemedicine adoption can lower morbidity, enhance preventive care, save time and resources, and relieve tertiary healthcare centers. While our focus was pediatrics, this model can extend to other age groups and conditions, but it should complement, not replace in-person care.

## Figures and Tables

**Table 1:** Status of the various clinical variables of participants.

Variable (*N* = 79)	Category	Count	Percentage
(1) Gender	Female	41	51.9
	Male	38	48.1
(2) Clinical history			
(a) Recent hospitalization	Yes	0	0
	No	79	100
(b) Chronic illness	Yes	8	10.1
	No	71	89.9
(c) Past drug history	Yes	6	7.6
	No	73	92.4
(3) Need of emergency care at presentation	Yes	7	8.9
	No	72	91.1
(4) Management			
(a) Health education	Given	79	100
	Not given	0	0
(b) Treatment	Given	77	97.5
	Not given	2	2.5
(c) Referral	Yes	33	41.8
	No	46	58.2

**Table 2:** Association between clinicodemographic profile and clinical outcome after 1 week.

Characteristics	Symptoms		Chi-square	*p* value	Crude odds ratio
	Resolved	Worsened			
Need of emergency care					
Yes	3	4	9.24	0.002	9.3 (1.78-48.68)
No	63	9			
Treatment					
Given	64	13	0.40	0.52	1.2 (1.08-1.33)
Not given	2	0			
Referral					
Yes	23	10	7.90	0.005	6.2 (1.55-24.92)
No	43	3			

## Data Availability

The raw data for this study can be shared.

## Abbreviations

TMC: Telemedicine center
AIIMS: All India Institute of Medical Sciences
RMO: Registered medical officer
SPSS: Statistical Package for Social Sciences
SD: Standard deviation
RR: Relative risk
CI: Confidence interval
VHT: Village health team.
